# Unintentional Injuries and Violence among Adults in Northern Jordan: A Hospital-Based Retrospective Study

**DOI:** 10.3390/ijerph14040343

**Published:** 2017-03-24

**Authors:** Manal M. Alzghoul, Mohammed K. Shakhatreh, Nihaya Al-sheyab

**Affiliations:** 1 School of Nursing, Faculty of Health & Behavioural Sciences, Lakehead University, 955 Oliver Road, Thunder Bay, ON P7B5E1, Canada; 2 Faculty of Science and Arts, Department of Mathematics & Statistics, Jordan University of Science and Technology, P.O. Box 3030, Irbid 22110, Jordan; mkshakhatreh6@just.edu.jo; 3 Faculty of Nursing, Jordan University of Science and Technology, P.O. Box 3030, Irbid 22110, Jordan; nasheyab@just.edu.jo

**Keywords:** unintentional injury, hospital-based, retrospective study, violence, Jordan, Jordanian

## Abstract

Injuries (unintentional and intentional) are the main cause of death and disability worldwide, including Jordan. The main purpose of this hospital-based retrospective study was to identify characteristics, causes, and risk factors of unintentional injuries and violence among all adult patients who approached the Accidents and Emergency department because of injury in Northern Jordan. Data were collected retrospectively from four major hospitals from January 2008 to January 2013. A total of 2425 Jordanian individuals who accessed and were treated by the four hospitals were included in this study. The findings show that the majority of patients who approached the Accidents and Emergency departments in the four hospitals were males (n = 2044, 87.16%) versus females (n = 301, 12.8%). Violence was the most common reason of injury (70.66%), followed by road traffic crashes (23.21%). The most common anatomical locations of reported injuries were the head (38.74%), followed by abdomen/pelvis and lower back, among males and females (9.93%). Violence had a high significant effect on the site of injuries. Patients who had been injured to the head because of a stab wound or fighting were substantially over-involved in head injuries, with injury rates 3.88 and 7.51 times higher than those who had been injured to the head due to gunshot, respectively. Even patients who had been injured to the head because of assault show much higher involvement in injury risk than non-assault patients (Odds Ratio = 8.46). These findings highlight the need for a large national study to confirm the findings. It also draws attention to the importance of public awareness and to special injury prevention programs that not only focus on saving lives and lessening the number of injuries, illnesses, and fatalities, but also to limit the social and economic burden of injury among adults in Northern Jordan.

## 1. Introduction

Injuries (unintentional and intentional) are the main cause of death and disability worldwide. [1,2,3,4]. Not only are injuries the main cause of death of five million people every year, which accounts for 9% of the world’s total annual death rate [5], but are also considered major causes of long-term disability where people with even a minor injury can remain disabled for at least one year after the incident [6].

Injuries are often caused by voluntary or involuntary exposure to thermal, mechanical, electrical, or chemical agents which result in minor or major tissue damage or dislocation. Injury is often a sudden, life-threatening incident with an extreme influence on the patient and their families’ physical, psychological, social, and emotional life [7,8,9,10]. 

There are several mechanisms for injuries, including unintentional injuries resulting from motor vehicle crashes, falls, poisoning, flames, or drowning, and intentional injuries, resulting from attempted suicide, attempted homicide, legal or court intervention, war, and other unspecified reasons [10,11].

In Jordan, the majority of the published literature identified road traffic crashes as the main cause of injury among adults. Over the last decade, road traffic crashes alone were responsible for the death of 7869 Jordanians and the injury of 171,143 individuals [12]. In 2007, traffic crashes in Jordan were classified as the second leading cause of death [13] of which children and young people represented more than 43% of those killed or seriously injured in crashes, compared to the 30% of worldwide corresponding percentage. The registered traffic crashes in 2007 were 110,630, resulting in 992 deaths and 17,969 injuries, with a financial cost of 280 million Jordanian Dinar (JOD) [14]. All of these studies show the significance of road traffic crashes as the main cause of injury among children, young adults, and the elderly [15]; however, most of these studies were mainly conducted by scholars in civil engineering and focused only on road traffic crash-related injuries. 

On the other hand, violence was identified as one of the main risk factors to death, disease, and disability, globally. In a recent report by the World Health Organization (WHO), about 4400 people lose their life every day because of intentional injury of a self-directed act or interpersonal violence [5]. In Jordan, deaths due to violence-related injuries are a public concern, yet, few studies have investigated violence in Jordan. A recent study conducted to describe patterns of gunfire violence in Northern Jordan found that males embraced the majority of firearm fatality cases, whereas females are rarely involved. Alcohol and substance abuse do not appear to play a role in violence-related injuries in Northern Jordan [16].

Nevertheless, to date there are no studies conducted in Jordan to specifically identify the characteristics, causes, or risk factors of different types and mechanisms of unintentional injuries and violence. It is of paramount importance to find out who is at risk among Jordanian adults and what the impact of injuries is on their bodies. Therefore, the main purpose of this study was to identify the characteristics, causes, and risk factors of unintentional injuries and violence among all adult patients who approached the accidents and emergency department because of injury in Northern Jordan.

## 2. Methods 

### 2.1. Design

A five-year, descriptive, cross-sectional, hospital-based retrospective study was conducted [17].

### 2.2. Setting, Sample, and Procedure

Four major representative hospitals were sought to conduct the study and gather relevant information in which all injured patients seek medical attention or are brought by the ambulance services in Northern Jordan. The estimated served population in these hospitals were 1.162 million people (Census, Jordan, 2013) [18].

Any adult patient (age 18 or older) who approached the Accidents and Emergency Department and created admission medical files in any of the four different hospitals in Northern Jordan due to injury from January 2008 to January 2013 was included in the current study. In this study, intentional injuries that include suicide, legal or court intervention, and war were excluded from the study and all death-related injury patients who were pronounced dead on arrival to the hospital were excluded.

A retrospective review was conducted using police records and electronic medical records of all adult (18 years and older) patients who accessed the Accidents and Emergency department (A&E) in the four major hospitals as a result of injury between January 2008 to January 2013. The list of legal cases obtained from police records, in which the main reason for seeking medical help was injury, regardless of type, was tracked down to identify adult patients’ medical records. When eligible patients were determined, the hospitals’ databases were searched for the medical number and the full information of each patient. Police records were used to retrieve the patient’s medical records because in Jordan it is mandatory to report any injury (unintentional and intentional) to the police if they approach the A&E department in any hospital and, usually, a police office is located at each A&E department for that purpose. Thus, the investigators realized that police records were the only possible way to include all injury patients. Police records are an accepted way to collect fatality and injury data in the Mediterranean region [19,20]. 

The investigators reviewed all adult patients’ files over the study period. These included daily medical notes/records of health care providers, including nurses, medical doctors, and surgeons, and all notes related to medical history. Each patients’ medical file was scanned for the following: biographical data (gender and age of patient at the time of injury), demographic characteristics (place of residency, occupation, educational level, smoking status, regular medication use, marital status, and socioeconomic status), medical history, previous hospitalization, presence of chronic diseases, mental or psychological disorders, epilepsy, hyperactivity disorder, autism, history of physical abuse, sensory motor impairment, current legal medication, and history of alcohol or drug addiction.

An injury tracking checklist was developed, according to the literature and the International Classification of Diseases version 10 (ICD-10) codes and external causes of injury [11], of which the following information were recorded for injuries: the type and anatomical location, the number, mechanism or the cause, the outcomes of each injury related hospitalizations, including surgery and disability, the number of previous injury-related hospitalizations, and the number of days hospitalized. 

### 2.3. Data Analysis

Descriptive statistics were used to present and describe all variables included in the analysis of the data for this study. More specifically, frequency tables and cross tables were constructed for age, gender, mechanisms of injury, and site of injuries. The aim of this study was to determine the effect of several covariates, including patient characteristics (age, gender, level of education) and mechanisms of injury (falling down, road traffic crashes, poison, contact with venomous animals and plants (bites/stings), violence, exposure to smoke/exposure to inanimate and mechanical forces) on the binary dependent variable (whether the patient had an injury or not).

Multiple logistic regression was used to measure the effect of these predictors on the dependent variable by calculating the odds ratio (OR) within the 95% confidence intervals (95% CI). A restriction was imposed on the set of the predictors that can be used in the analysis. Each candidate predictor was required to have a data value rate of at least 70% or higher. This constraint was applied for computational stability purposes during the process of model estimation. After applying this restriction, only age, gender, and violence were retained. Separate analyses were performed for each site injury. We have performed separate analyses for all types of injuries on age, gender, and violence, and only the three models included in the paper showed a significant effect.

The statistical package for social scientist (SPSS) software version 17 (SPSS Inc., Chicago, IL, USA) was used to analyze the data in this study.

### 2.4. Ethical Considerations

The study was approved by the Institutional Review Board at Jordan University of Science and Technology, the Jordanian Ministry of Health and the police department in Irbid city (Project code (177/2012)). All data were kept confidential and access to data was only granted to the principal investigator and trained research assistants who helped in data collection.

## 3. Results

A total of 2425 Jordanian individuals who accessed and were treated by the four hospitals were included in this study. This number is subject to the number of patients who have been injured, the type of injury, or missing observations. Table 1 represents the distribution of patients at the time of injury according to age and gender. The age ranged from 18 to 56 years (mean = 31.53, SD = 12.647). The majority of injuries occurred in the male population and, specifically, in the age group of 18–27 years old, followed by the age group of 28–37 years old, which was also the age group with the highest risk among females, as well.

In the male age group of 28–37 years old, 176 experienced one injury, 79 suffered two injuries, 23 suffered injuries, only one suffered four different injuries, and four suffered a total of five injuries. In the same age group for females (28–37 years old), 28 suffered one injury, 15 suffered two injuries, five suffered three injuries, only one suffered four different injuries, and two suffered a total of five different injuries. Male patients (2044, 87.16%) outnumbered females (301, 12.8%) (Table 1). 

Table 2 represents the distribution of injury types by cause of injury. People, both males and females, were most likely injured through violence (70.66%) followed by road traffic crashes (23.21%). Fighting, stab wounds, gunshot, and assault were the main reasons identified as reasons of violence. There were some similarities, but also several differences between male and female patients regarding the site of injuries (Table 3). In both males and females, the head was the most common site of injury (127 in females, 962 in males), followed by the abdomen/pelvis and lower back (38 females, and 241 males). However, in male patients, the third-most common injury occurred to the wrist (212), followed by injuries to the knees and lower legs (186). On the other hand, in females, the second-most common injury occurred to the abdomen and lower back (38), followed by the shoulder and upper arm (30). 

Descriptive characteristics of patients’ injuries in the head, hips, and thigh, and knee and lower legs in Northern Jordan are presented in Table 4, Table 5 and Table 6. All of these three types of injuries are major problems among 18–27 year-old patients. Approximately 53.68% of head injuries, 57.75% of hip and thigh injuries, and 58.68% of knee and lower leg injuries involve 18–27 year-old patients, whereas the corresponding rate is 55.46% of non-accidental head injuries, 54.38% of non-accidental hip and thigh injuries, and 54.25% of non-accidental knee and lower legs injuries. The majority of patients who had an injury to one of these types of injuries were men. Violence is another factor for causing injuries. In this study, in injuries that occurred because of fighting 86.19% had head injuries, 57.76% had hip and thigh injuries and 75.76% had knee and lower leg injuries. Similarly, injuries that occurred because of assault were head injuries (4.52%), hips and thigh injuries (11.27%), and knee and leg injuries (3.03%). Moreover, injuries that occurred because of gunshot were head injuries (0.9%) and hips and thigh injuries (19.72%). Furthermore, injuries that occurred because of stab wounds were head injuries (8%), and hip and thigh injuries (8.45%). Injuries that occurred because of burning formed the smallest rate among the three types of injuries.

Table 7 provides the results of fitting three multiple logistic regression models of the site of injuries, namely, injuries to the head, injuries to hips and thigh, and injuries of the knee and lower legs, against age, gender, and violence, separately. All three models showed that gender and age were not significant and, therefore, they were excluded from the analysis, whereas these models revealed that violence had a high significant effect on the site of injuries. Patients who had suffered injury to the head because of a stab wound or fighting are substantially over-involved in head injuries, with the injury risk being 3.88 and 7.51 times higher than those patients who had been injured to head due to a gunshot, respectively. Even patients who had suffered injury to head because of assault showed significantly higher injury risk than non-assault patients (OR = 8.46). Contrary to injuries to head, patients who had been injured to the hips and thigh because of a stab wound or fighting had substantially low involvement in hip and thigh injuries, with an injury risk of 0.091 and 0.089 times lower than those patients who had suffered injury to the hips and thigh because of a gunshot, respectively. However, patients who had suffered injury to the hips and thigh because of assault also showed lower involvement of injury risk than non-assault patients (OR = 0.36). Similarly, patients who had been injured in the knee and lower legs because of a stab wound or fighting or assault were substantially less involved in knee and lower legs injuries, with an injury risk of 0.265, 0.27, and 0.21 times lower than those patients who had been injured to the hips and thigh because of a gunshot, respectively. 

## 4. Discussion

The current study is the first to report the characteristics, causes, or risk factors of different types and mechanisms of unintentional injuries and violence among Jordanian adult patients who were admitted to the hospital because of injuries in Northern Jordan. The most common anatomical locations of reported injuries were the head, followed by abdomen/pelvis and lower back. The findings of this study showed that males were at significantly higher risks of injuries compared to females in most age groups. Additionally, the findings highlight that being young (18–27) has exposed patients to higher risk for injury. These findings are congruent with similar previous studies [15,21,22,23]. 

The current study shows that violence is the main reason of injury, followed by motor vehicle crashes. This finding is very important because it is the first in Jordan to demonstrate violence as a main cause of injury in Jordanian adults. In fact, most previous studies demonstrated that motor vehicle crashes were the most common reason of injury in the country [24]. 

The relationship between violence and injury has been well documented in many countries [1,5,25,26,27,28]. Findings from these studies show that males and young adults were at the highest risk of violence-related injury, which is consistent with the current study.

The current study found that motor vehicle crashes are the second-leading cause of injury in the study population. These findings are congruent with local studies in Jordan. In 2007, traffic crashes were classified as the second-leading cause of death among Jordanians [13] (110,630 traffic crashes were registered, resulting in 992 deaths and 17,969 injuries). It was also found that the majority of driver and passenger casualties (both fatalities and injuries) belonged to the age group of 18–42 years, whereas the highest percentage of pedestrian casualties belonged to the age group of 0–18 years [14]. Previous retrospective studies in Jordan identified motor vehicle crashes as the most common cause of traumatic spinal cord injuries (44.4%) followed by bullet injuries (28.8%) [29]. However, in comparison with the current study, findings should be made with caution because most of these particular studies were conducted by professionals outside the health sector with no emphasis on identifying the risks of injury in the Jordanian population, but mainly to identify the reason of the traffic crashes from a civil engineering perspective. 

In regard to the site of injury, the current study found that the most common anatomical locations of injuries were the head, followed by abdomen/pelvis and lower back. Few studies about the site and the cause of injury have been conducted in Jordan. Shotar et al. [15] found that road traffic crashes affect children and adolescents and mainly impacts their head, neck, and upper extremities. However, in another study that looked for the pattern of firearm fatalities in Northern Jordan, the chest was the most frequent site for bullet injuries in male adolescents and young adults [16]. Additionally, an earlier study found that during 2004 stabbing by knife was the cause in 24 patients, gunshot in one, and falling on a sharp object in another, whereas 26 patients were managed (25 males and one female), and the mean age was 22.5 years [30]. Unlike the current study, these studies did not investigate the relationship between injury and violence, nor did they compare violence to other causes of injury.

Furthermore, the current study found that patients who had suffered head injuries because of stab wounds or fighting were substantially over-involved in head injuries with the injury risk significantly higher than those who had suffered injuries to the head due to gunshot, respectively. This can be explained by the fact that most of people who are shot with a gun in the head are more likely to die [16], as compared to those who suffer an injury to the head because of fighting. Another explanation is that the use of guns is still limited in Jordan; thus, the incidence of such injuries is minimal.

Finally, the current study found no significant relationship between chronic illness and injury. One explanation for this could be because that most of the study sample was young and only a few of them were diagnosed with chronic illnesses; thus, statistical analysis was limited.

### Limitations

While this study provides one of the first reports on adult injuries in Northern Jordan, it has some limitations. Firstly, not all medical files were found because, although all patients who approached the emergency department with injuries were reported to the police, only patients who were admitted to the hospital generated medical files. If a patient was not admitted to the hospital and discharged directly after being treated in the emergency department, no medical file existed for that patient; therefore not all information sought was retrieved. Secondly, the absence of electronic medical records in two major hospitals, and the lack of a database to record and store this information, made it very challenging to find patient files in allocated storage rooms. Additionally, there were missing data in the patient medical files; not all information that was sought was well-recorded, including a lack of socio-demographic characteristics and medical history, and this limited the data analysis. Furthermore, there were missing information in the medical folders of admitted patients due to lack of standardization in reporting nursing and medical notes across hospitals and the lack of documentation of all relevant information about patients in their records. A solution is needed, such as using an injury registry system and mandatory recording of all socioeconomic data, to deal with the lack of recording all pertinent information in order to document and report the accurate significance of the problem. Thirdly, the design was cross-sectional and retrospective, thus, the data retrieved could not imply a causal relationship. Finally, the study was conducted in Northern Jordan only and this could limit the generalizability of the findings to other parts of Jordan.

## 5. Conclusions

Males were at the highest risk for injury in Northern Jordan, and violence was the main cause of injury, followed by the motor vehicle crashes. These findings highlight the need for further studies to explore this area in more depth and improve our understanding of the scope and characteristics of violence-related injury to be able to develop appropriate interventions to prevent such types of injuries.

Despite the study limitation of being a hospital-based study, and challenges in collecting the data, this study is important because it offers starting point data and the possibility to understand injuries, their risk factors, their characteristics, and to assist in identifying people who are at high risk. Identifying factors of adult injuries is necessary for the development of successful prevention strategies at a national level that minimizes or prevents these risks. It also provides a valuable baseline for identifying important areas for future research.

## Figures and Tables

**Table 1:** Distribution of Jordanian adult patients at the time of injury according to age and gender (n = 2345).

		Age of Patient at the Time of Injury					
		18–27	28–37	38–47	48–57	58+	Total
Gender	Female	116	76	53	22	34	301
	Male	1061	523	263	109	88	2044
	Total	1177	599	316	131	122	2345

**Table 2:** The mechanisms (causes of injuries) of injuries in Jordanian adult patients (n = 2345). Frequency (proportion %) injured by mechanisms of injury.

Mechanism	Female		Male		Total	
	Frequency	%	Frequency	%	Frequency	%
Falling Down	23	8.85	60	3.23	83	3.92
Road traffic crashes	84	32.31	408	21.94	492	23.21
Poison	6	2.31	6	0.32	12	0.57
Contact with venomous animals and plants (Bites/stings)	6	2.31	10	0.54	16	0.75
Violence	139	53.46	1359	73.06	1498	70.66
Exposure to smoke, Exposure to inanimate	2	0.77	7	0.38	9	0.42
mechanical forces	0	0	10	0.54	10	0.47

**Table 3:** Description of injury sites of Jordanian adult patients by gender (n = 2811). Frequency (Proportion %) injured by gender.

Site of Injuries	Female		Male		Total	
	Frequency	%	Frequency	%	Frequency	%
Head	127	36.60	962	39.04	1089	38.74
Neck	13	3.75	112	4.55	125	4.45
Thorax	21	6.05	179	7.26	200	7.11
Abdomen, lower back	38	10.95	241	9.78	279	9.93
lumbar spine and pelvis						
Shoulder and Upper Arm	30	8.65	152	6.17	182	6.47
Elbow/Forearm	22	6.34	164	6.66	186	6.62
Wrist/Hand	25	7.20	212	8.60	237	8.43
Hip and Thigh	29	8.36	118	4.79	147	5.23
Knee and Lower Leg	21	6.05	186	7.55	207	7.36
Ankle/Foot	8	2.31	61	2.48	69	2.45
Multiple Regions of Body	6	1.73	34	1.38	40	1.42
Spin	1	0.29	9	0.37	10	0.36
Internal Organs	6	1.73	34	1.38	40	1.42
Total	347	100	2464	100	2811	1.42

**Table 4:** Jordanian adult patients characteristics of having injury to head, or not, in Northern Jordan (n = 1498).

	Injury to Head			
	No (n = 723)		Yes (n = 775)	
Patient Characteristics	n	%	n	%
*Gender*				
Male	658	91.01	701	90.45
Female	65	8.99	74	9.55
*Age*				
18–27	401	55.46	416	53.68
28–37	182	25.17	197	25.42
38–47	93	12.86	91	11.74
48–57	22	3.04	50	6.45
58+	25	3.46	21	2.71
*Violence*				
Fighting	550	76.07	668	86.19
Burning	5	0.69	3	0.39
Stab wound	100	13.83	62	8
Gun-shot	43	5.95	7	0.9
Assault	25	3.46	35	4.52

**Table 5:** Jordanian adult patients’ characteristics of having injury to hips and thigh, or not, in Northern Jordan (n = 1498).

	Injury to the Hip and Thigh			
	No (n = 1427)		Yes (n = 71)	
Patient Characteristics	n	%	n	%
*Gender*				
Male	1296	90.82	8	11.27
Female	131	9.18	63	88.73
*Age*		0		0
18–27	776	54.38	41	57.75
28–37	358	25.09	21	29.58
38–47	177	12.4	6	8.45
48–57	70	4.91	3	4.23
58+	44	3.08	2	2.82
*Violence*				
Fighting	1177	82.48	41	57.75
Burning	6	0.42	2	2.82
Stab wound	156	10.93	6	8.45
Gun-shot	36	2.52	14	19.72
Assault	52	3.64	8	11.27

**Table 6:** Jordanian adult patients characteristics of having injury to the knee and lower legs, or not, in Northern Jordan (n = 1498).

	Injury to the Knee and Lower Leg			
	No (n = 1399)		Yes (n = 99)	
Patient Characteristics	n	%	n	%
*Gender*				
Male	1267	90.56	92	92.93
Female	132	9.44	7	7.07
*Age*				
18–27	759	54.25	58	58.59
28–37	357	25.52	24	24.24
38–47	174	12.44	9	9.09
48–57	70	5	2	2.02
58+	39	2.79	6	6.06
*Violence*				0
Fighting	1143	81.7	75	75.76
Burning	7	0.5	1	1.01
Stab wound	152	10.86	10	10.1
Gun-shot	40	2.86	10	10.1
Assault	57	4.07	3	3.03

**Table 7:** Results of multiple logistic regression analysis in regards to the site of injury among Jordanian adult patients (n = 1498).

Odds Ratio (OR) within the 95% Confidence Intervals (95% CI)					
Site Injury		Odds Ratio	Lower	Upper	Sig.
Injuries to Head					
	Violence				
	Fighting	7.512	3.358	16.806	0.0000
	Burning	3.771	0.733	19.414	0.1120
	Stab wound	3.882	1.648	9.149	0.0020
	Assualt	8.462	3.288	21.776	0
Injuries to Hips and Thigh					
	Violence				
	Fighting	0.089	0.045	0.174	0.0000
	Burning	0.8	0.145	4.423	0.798
	Stab wound	0.091	0.033	0.25	0.0000
	Assualt	0.362	0.139	0.943	0.0380
Injuries to Knee and Lower Legs					
	Violence				
	Fighting	0.274	0.132	0.568	0.0000
	Burning	0.586	0.064	5.32	0.6350
	Stab wound	0.265	0.103	0.678	0.006
	Assualt	0.212	0.055	0.819	0.024
